# Fellows and Observers Are Not a Problem for Infection in the Operating Rooms of Teaching Centers

**DOI:** 10.3390/tropicalmed6020043

**Published:** 2021-03-31

**Authors:** Verónica Montiel, Daniel Pérez-Prieto, Simone Perelli, Joan Carles Monllau

**Affiliations:** 1 Orthopedic Surgery and Traumatology Department, Clínica Universidad de Navarra, Av. Pio XII, 36, 31008 Pamplona, Navarra, Spain; vmontiel@unav.es; 2 Orthopedic Surgery and Traumatology Department, Hospital del Mar, Passeig Marítim de la Barceloneta, 25, 29, 08003 Barcelona, Cataluña, Spain; sperelli@hospitaldelmar.cat (S.P.); jmonllau@hospitaldelmar.cat (J.C.M.); 3 Hospital Universitari Dexeus- Grupo Quirónsalud, Carrer de Sabino Arana, 5, 19, 08028 Barcelona, Cataluña, Spain

**Keywords:** overcrowded operation room, teaching hospitals, intraoperative complications, postoperative complications, infection

## Abstract

Purpose: The aim of the present study was to determine whether the risk of complications increases with the number of people in the operating room (OR). Several studies have stated that an increased number of people in the OR increases not only the risk of infection but also the risk of intraoperative complications due to distractions during the surgery. Materials and Methods: This retrospective study included all patients who had surgery between January 2017 and January 2018 in an OR with the usual surgical team and three or more observers. Patient demographic data, surgical details (duration of the surgery, the surgery being open or arthroscopic, and whether a graft was used), and intraoperative and postoperative complications were recorded. Results: A total of 165 surgeries were recorded, with a mean operating time of 70 min (40% open surgeries, 37% arthroscopic surgeries, and 23% combined open and arthroscopic procedures). The main intraoperative complications were vessel damage, nerve damage, premature cement setting, and leg-length discrepancy, with 1 case each. The main postoperative complications were rigidity (8 cases), unexplained pain (11 cases), failed meniscal suturing (3 cases), a postoperative stress fracture (1 case), correction loss in osteotomy (1 case), and wound problems not related to infection (1 case). There were no cases of infection. Discussion: The present study shows that the complication rate when having observers in the OR is comparable to the reported data. The key to avoiding complications is for everyone to comply with basic OR behavior.

## 1. Background

An increased number of people in the operating room (OR) has been hypothesized as a cause for intraoperative disruptions in several papers. The suggestion is that it leads to an increase in intraoperative complications, heightens the risk of infection, as well as the risk of intraoperative complications, due to distractions during the surgery. The rate of complications has been stated as ranging from 3% to 22% by the World Health Organization [1], half of which could have been prevented. A few studies have stated that OR sizes are inadequate [2]. This can be especially important for orthopedic procedures in which the appliance burden is quite substantial [3]. This has led to new designs with increased OR sizes to accommodate more equipment and larger teams [3]. However, few changes have been made to improve safety in the OR. Many studies highlight the importance of accidental surface contamination, excessive traffic, loud noises, poor furniture and equipment, or uncomfortable working environments (temperature, humidity, air quality) in term of effects on the outcome of the surgery [3,4]. Some studies have also considered the need to exit the OR in order to bring essential items into the OR once surgery has started and the number of unnecessary door-opening events in their analysis [5,6]. Other authors have emphasized the importance of understanding the task at hand and the movements within the different areas of the OR to avoid accidental contamination or interference during the surgery [7,8]. Even loud sounds have been demonstrated to have a negative impact in the OR. They are considered relevant in terms of the increased stress generated that effects the team’s ability to perform tasks safely and efficiently [9,10,11,12,13,14,15,16]. In the literature, the theory is that having many people in the OR increases noise and the rate of accidental contamination [17,18]. Moreover, the CDC guidelines from 1999 recommend limiting the number of people in the OR [19]. This assertion could directly affect teaching hospitals at the residency training level and in fellowship positions, and could equally limit the number of observers.

The aim of the present study was to determine whether the risk of complications during orthopedic procedures increases with the number of people in the OR. This assertion could directly affect how teaching hospitals are organized. The hypothesis was that an overcrowded OR would not increase complications.

## 2. Methods

This was a retrospective cohort study that included all patients that had more than 3 or more scrubbed surgeons, one scrubbing nurse, one circulating nurse, an anesthesiologist, and 3 or more observers during their surgery between January 2017 and May 2018. All observers were residents, fellows, or visiting faculty. This study was approved by the local committee (11/145/1350). All patients underwent surgery in the same hospital with the same surgical team. The surgical details recorded included the duration of the surgery, it being an open or arthroscopic surgery, and whether any kind of graft was used. All types of intraoperative and postoperative complications were also registered. 

Infection was defined according to the Pro-Implant Foundation criteria [20]. In that sense, prosthetic joint infection (PJI) was defined as when at least one of the following criteria was present: (i) visible purulence of the aspiration or at the surgical site; (ii) the presence of a sinus tract communicating with the implant; (iii) acute inflammation in histopathology sections of intraoperative tissue (as determined by the pathologist); (iv) acute inflammation in preoperative joint aspiration (leukocyte count of >2.000 or >70%); (v) confirmatory microbial growth in synovial fluid, periprosthetic tissue, or sonication culture.

A minimum follow-up of 2 years was requested for all included patients. A sample size calculation was made. 

Accepting an alpha risk of 0.05 and a beta risk of 0.2 in a two-sided test, 160 subjects were necessary to recognize a difference greater than or equal to 3% of complications; taking into account that the complications analyzed here have an incidence reported rate of 1%, a drop-out rate of 10% was anticipated. The statistical analysis was done using the SPSS 18.0 (SPSS Inc., Chicago, IL, USA) statistical package.

## 3. Results

A total of 165 patients were included (165 surgeries). The mean age was 43 and 56% were male and 44% were female (Table 1). The main surgeries performed were anterior cruciate ligament (ACL) arthroscopic reconstruction, osteotomies, total knee arthroplasties, and unicompartmental knee arthroplasties (Table 2). The mean operating room time was 70 min. Thirty-seven percent of the reviewed surgeries were open surgeries, 33% were arthroscopic surgeries, and 30% of the surgeries combined open and arthroscopic procedures (Table 2). Among the open surgeries, the most common were osteotomies (11% of all surgeries), total knee arthroplasties (10% of all surgeries), unicompartmental knee arthroplasties (5% of all surgeries), and hardware removal (3%). Regarding the arthroscopic surgeries, the most common were meniscectomies (15% of all surgeries), meniscal sutures (5% of all surgeries), and arthroscopic arthrolysis (5% of all surgeries). The most common procedures combining open and arthroscopic approaches were ACL reconstructions (22% of all surgeries), ACL revisions (7% of all surgeries), and meniscal transplants (2% of all surgeries). No graft was used in 62% of the patients, an autograft was used in 12% of the patients, an allograft was used in 24% of the patients, and a combination of auto- and allografts was used in 3% of the patients. Only 2% of the patients were smokers. The total rate of complications (intraoperative and postoperative) was 17% (29 cases) (Table 3). The main intraoperative complications were vessel damage in 1 case (0,6%), nerve damage in 1 case (0.6%), premature cement setting in 1 case (0.6%), and leg-length discrepancy in 1 case (0.6%). The main postoperative complications were rigidity in 8 cases (4.8%), unexplained pain in 11 cases (6.6%), failed meniscal suturing in 3 cases (1.8%), stress postoperative fracture in 1 case (0.6%), correction loss in osteotomy in 1 case (0.6%), and wound problems not related to infection in 1 case (0.6%). There were no cases of surgical site infection (SSI).

## 4. Discussion

The present study shows that the complication rate when having observers in the OR is comparable to the reported data, which is known to be less than 1% for vessel and nerve damage, altered wound healing, leg-length discrepancy, and bone fractures. The rates ranged from 2% to 5% for pain and rigidity in total knee arthroplasties [21]. The infection rate in total knee arthroplasties has been put at around 2%. The complication rates for osteotomies have been reported to be 2% to 5% for infection, 2% to 5% for hematoma, 1% to 4% for thrombosis, 2% for correction loss, 3% to 12% for aseptic nonunion or delayed union, 4% for 6% for intraoperative or postoperative lateral cortex fracture, 4% for delayed unions, 4% for symptomatic hardware, 4% for limited hardware failure, 6% for delayed wound healing, 10% for cellulitis, 3% for non-displaced lateral tibial plateau fractures and hematoma, 1% for increased tibial slope (≥10°), and 1% for stiffness [22,23,24]. Meniscal suture failure has been reported to occur in 5.5% of the cases [25].

Various studies have observed that several preventable errors that are unrelated to training, knowledge, or skill occur in the OR. They mostly represent cognitive or teamwork failures [4,26,27,28]. Wiegmann et al. demonstrated that the number of surgical errors have positive correlations with surgical flow disruptions and communication failures [29]. Moreover, several authors have proven that minor problems influence global intraoperative performance, increase the rate of major problems and the OR time, and diminish the capacity to correct or compensate for major errors [7,28,29,30,31,32]. Having five additional people in the OR on top of the necessary OR personnel has been reported as a risk factor for increased OR microbiological counts [33]. All of these factors could potentially be influenced by the number of people in the OR. However, the truth of the matter is that behavior in the OR is more important than the number of people inside it. An increased number of people in the OR has also been correlated with disruption of OR traffic. Other factor to consider are shift changes and unnecessary door opening. They have been proven to decrease the efficiency of ventilation systems [33] and increase microbiological counts in ORs [33,34,35], and therefore increasing the rate of surgical site infection (SSI) [36,37,38,39,40]. They may also distract the operating surgeon [36,37,38,39,41]. Again, these distractions should be considered poor OR practices that can be monitored and corrected through education and adherence to OR rules. Studies have reported variable numbers of door openings in orthopedic surgery, which range from 5 to 87 per hour [38,42] or 3 to 20 per surgery [43]. Some authors state that humans shed an important number of particles and skin fragments that can act as a reservoir for air contamination [44,45]. Moreover, the increase in SSI has been correlated to an increase in unnecessary door opening and avoidable OR flow (or movement in the OR) in some studies [36,37,38,41]. Door opening after skin incision only accounts for half (53%) of the total number of door openings during an OR session [46]. Most of these door openings are motivated by a need for supplies (23%), information (11%), or scrubbing (7%) [38]. Observers do not usually participate in any of these tasks. The circulating nurse (25%) or commercial agent (20%) causes most of the door openings. However, 47% of the door openings have been reported to be for unknown causes. These are the ones most easily preventable by reminding everyone in the OR to comply with correct OR behavior. We consider that setting strict rules as to when observers should enter the OR, to avoid going in and out of the OR for no reason, limiting OR flow, and restricting movement within the OR is key to controlling SSI when having many observers in the OR. 

Despite these results, Alizo et al. stated that increased OR traffic is not an independent risk factor for SSI. However, they found a clear relationship between operative time and traffic flow, which has been stated as a risk factor that increases the incidence of SSI [42]. Operative time has also been related to the number of communication failures [47]. Catchpole et al. emphasized the importance of understanding the task at hand and the OR flow within the different areas of the OR in preventing accidental contamination or interference during surgery [7,28]. Whether the number of communication failures is related to the number of people in the OR is still to be determined; nevertheless, it can be related to increased noise in the OR. When it comes to the low rate of infection in the present study, several considerations must be made. The most important is the aforementioned personnel training. Some other are the fact that arthroscopic procedures with a lower rate of infection are predominant in the present study. Lastly, the addition of the vancomycin technique in ACL procedures has been proven to reduce infection risk.

Noise has also been reported as a risk factor for increasing the SSI rate [17,18]. Kurmann also stated that conversations unrelated to the ongoing surgery are significantly louder [18]. It appears logical that if the number of people in the OR increases, the noise can also increase. Again, this is something that can be explained to visitors to avoid parallel conversations going on in the OR. Zehng et al. reported that most conversations do not delay or interrupt surgical workflow significantly and that the events that generate the most delay were changing instruments or waiting for one to become available (3.4 min/h) [48].

Alarcon et al. stated that laparoscopic surgery has a higher percentage of OR space occupied with instrumentation and results in multiple cables and tubes crossing the OR space perpendicularly, thereby impeding the circulation of personnel [49]. We did not observe a difference in complications between open and arthroscopic surgery. One reason might be how we placed the cables in a concentrated area of the OR and the instructions given to personnel and observers to avoid circulating in that area to prevent accidental contamination. Accidental contamination of surfaces can be reduced with adequate education of the OR team and visitors, which is a crucial point [36,37,38].

Engelmann et al. proved that introducing a number of behavioral rules to limit noise reduced the noise level originating from communications unrelated to the ongoing operation. They included avoiding private conversations in the background, banning mobile phones from the OR, keeping doors closed, and lowering equipment noise alarms significantly [17]. The key to preventing these complications is for everyone to comply with basic OR behavior by preventing the disruption of normal OR circulation, the unnecessary opening of doors, undetected contamination, and making excessive noise.

Studies analyzing the consequences of OR behavior for the patient are not without flaws. They often have no control group and measure a number of heterogeneous variables and points. Therefore, there is a high risk of bias in publications and of having confounding variables interfere with results and conclusions. This study was not without limitations. Firstly, this is a retrospective study. The number of patients recorded was low and may be insufficient to draw definitive conclusions regarding the possible complications caused by having many doctors in the operation room. This implies that it does not allow to analyze the different subgroups separately. Nonetheless, we think that having observers in the OR, when properly instructed as to when to enter the OR and where to place themselves, does not drastically increase complications, given the number of patients recorded. Additionally, there is no control group, because we considered that a comparison with the complication rate extensively and in detail described in the literature was the most correct way to analyze our data, especially considering that the complication rate found in our sample was as low as that reported in the literature. Moreover, we did not record the number of nurses, anesthesiologists, or other personnel in the OR. Lastly, we only recorded the number of people in the OR, but not the flow of personnel going in and out of the OR. Those comings and goings from the OR have been seen to lead to an increase in infections because they disrupt laminar flow, this is one other limitation that should be considered. 

Future studies should assess the rate of complications in a control group. Nevertheless, it would be interesting to evaluate how the COVID-19 pandemic restrictions in the OR have affected fellows’ surgical training. It would be also interesting to evaluate if these restrictions in the OR have reduced complications. 

## 5. Conclusions

The present study shows that the complication rate associated with having observers in the OR is comparable to the reported data, with no increase in the rate of infection or other postoperative and intraoperative complications.

## Figures and Tables

**Table 1:** Demographic Data.

Number of Patients (N)	165
Rate Women (%): Men (%)	72 (44%): 93 (56%)
Mean age (SD)	43 (18) years
Mean height in m. (SD)	1.7 (0.93) m
Mean weight in Kg. (SD)	73 (15) Kg
Body mass index (SD)	25 (4.05)
Smokers (%)	3 (2%)

**Table 2:** Types of Surgeries and Use of Allografts.

Mean Duration of the Surgery (SD)	70 (31) min.
Rate of open (%): arthroscopic (%): combined (%) procedures	61 (37%): 54 (33%): 50(30%)
Use of graft: No graft (%) Autograft (%) Allograft (%) Auto and allograft (%)	103 (62%) 19 (12%) 39 (24%) 4 (3%)
**Open procedures**	
Osteotomies	18 (11%)
Total knee arthroplasties	17 (10%)
Unicompartmental knee arthroplasties	8 (5%)
Hardware removal	5 (3%)
Total knee revision	1 (0.6%)
Anterolateral tenodesis	3 (2%)
Osteochondral fragment fixation	1 (0.6%)
Lateral patellofemoral ligament reconstruction	1 (0.6%)
Tendon procedures	7 (4%)
**Arthroscopic procedures**	
Meniscectomies	24 (15%)
Meniscal sutures	9 (5%)
Arthroscopic arthrolysis	9 (5%)
Cartilage procedure	2 (1%)
Tibial eminence fixation	3 (2%)
Cyst removal	2 (1%)
Synovectomy	4 (2%)
Osteochondral fragment fixation	1 (0.006%)
**Combined procedures**	
Anterior Cruciate Ligament (ACL) reconstructions	35 (22%)
ACL revisions	11 (7%)
Meniscal transplants	3 (2%)
Cyst removal	1 (0.6%)

**Table 3:** Complications.

Total complications (%)	17%
**Intraoperative complications**	
Vessel damage	1 case (0.6%)
Nerve damage	1 case (0.6%)
Premature cement setting	1 case (0.6%)
Leg-length discrepancy	1 case (0.6%)
**Postoperative complications**	
Rigidity	8 cases (4.8%)
Unexplained pain	11 cases (6.6%)
Failed meniscal suturing	3 cases (1.8%)
Stress postoperative fracture	1 case (0.6%)
Correction loss in osteotomy	1 case (0.6%)
Wound problems not related to infection	1 case (0.6%)
Infection	0 cases (0%)

## Data Availability

Not applicable.

## Abbreviations

OR	Operating Room
CDC	Centers for Disease Control and Prevention
ACL	Anterior Cruciate Ligament
SSI	Surgical Site Infection
